# Supplementary material for: A foundational vision transformer improves diagnostic performance for electrocardiograms
Source: NPJ Digit Med. 2023 Jun 6;6:108. doi: 10.1038/s41746-023-00840-9 (PMC10242218; doi:10.1038/s41746-023-00840-9)
Supplement: Supplementary file 2 — Supplemental Material [file 41746_2023_840_MOESM2_ESM.pdf]

Supplementary Figures

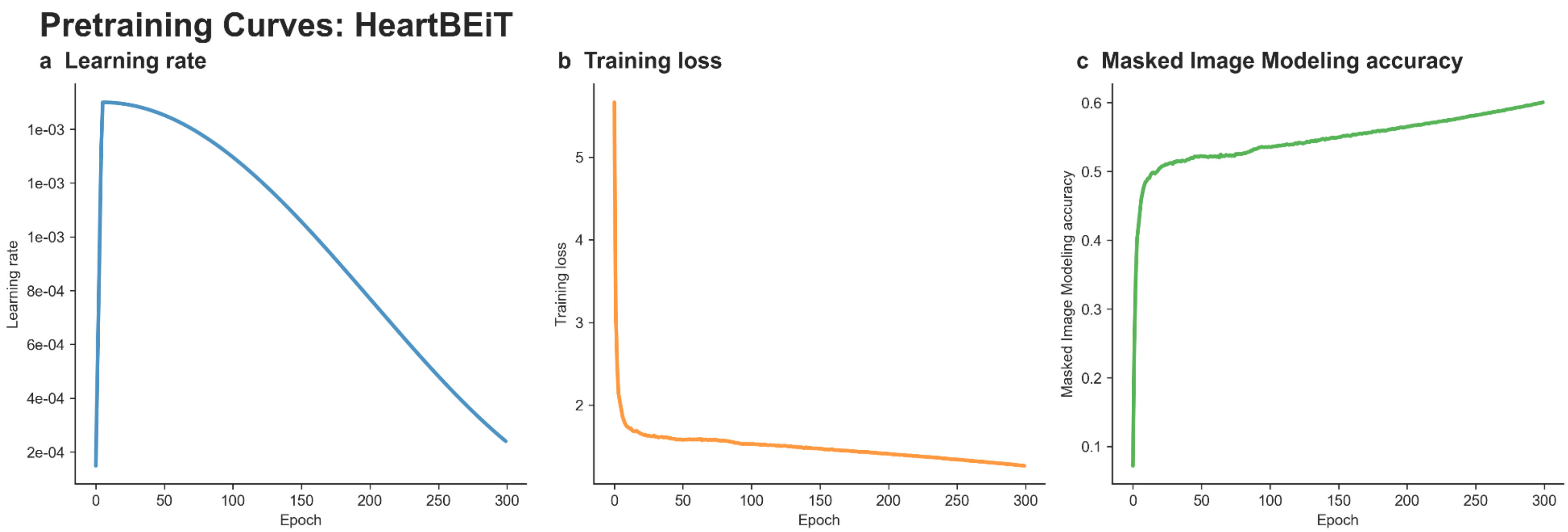

Supplementary Figure 1. Pretraining curves for HeartBEiT.

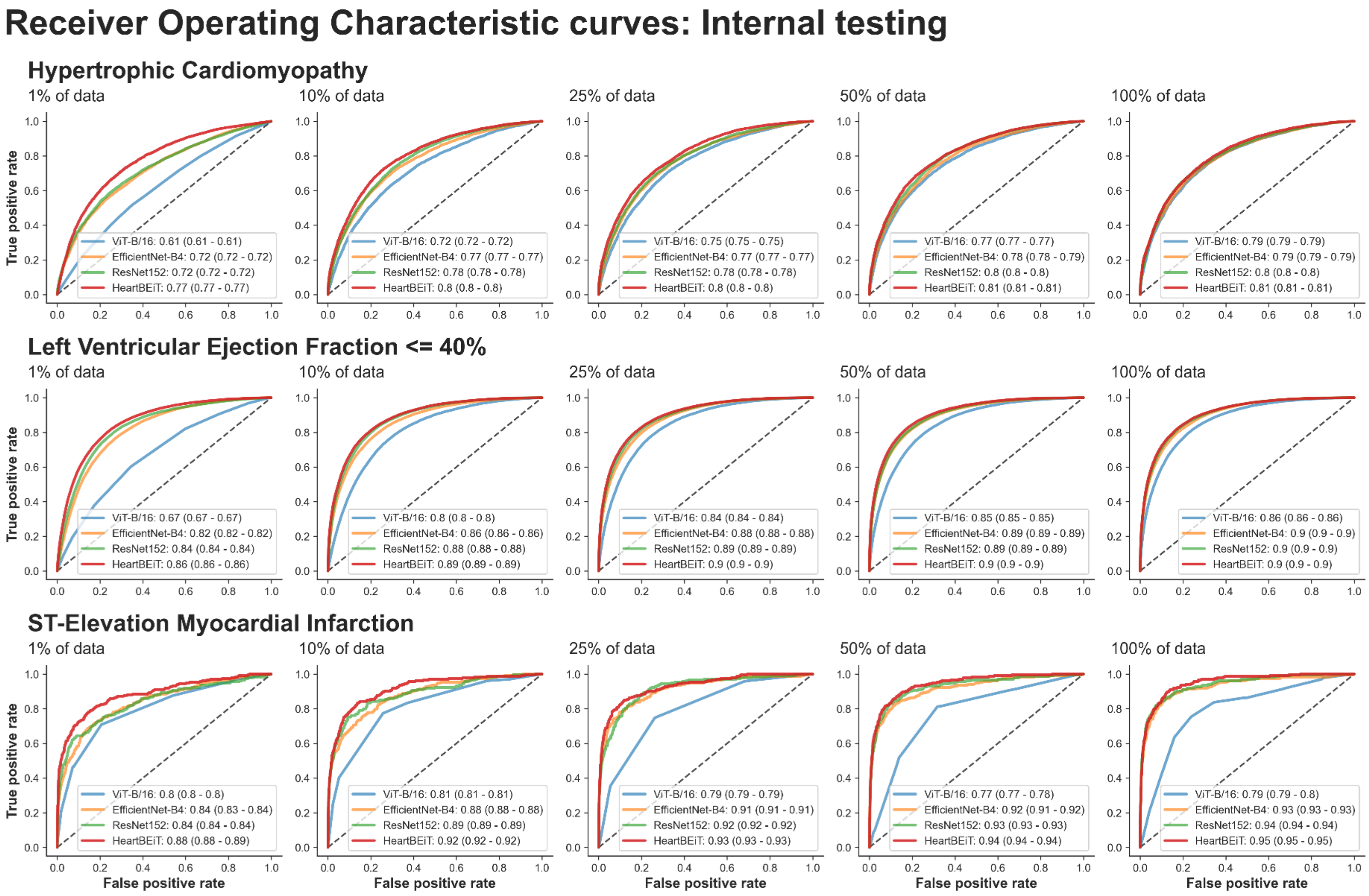

Supplementary Figure 2. Receiver Operating Characteristics Curves for Internal Testing of Vit-B/16, EfficientNet-B4, ResNet-152, and HeartBEiT for classification of Hypertrophic Cardiomyopathy, Left Ventricular Ejection Fraction ≤40%, and ST-Elevation Myocardial Infarction.

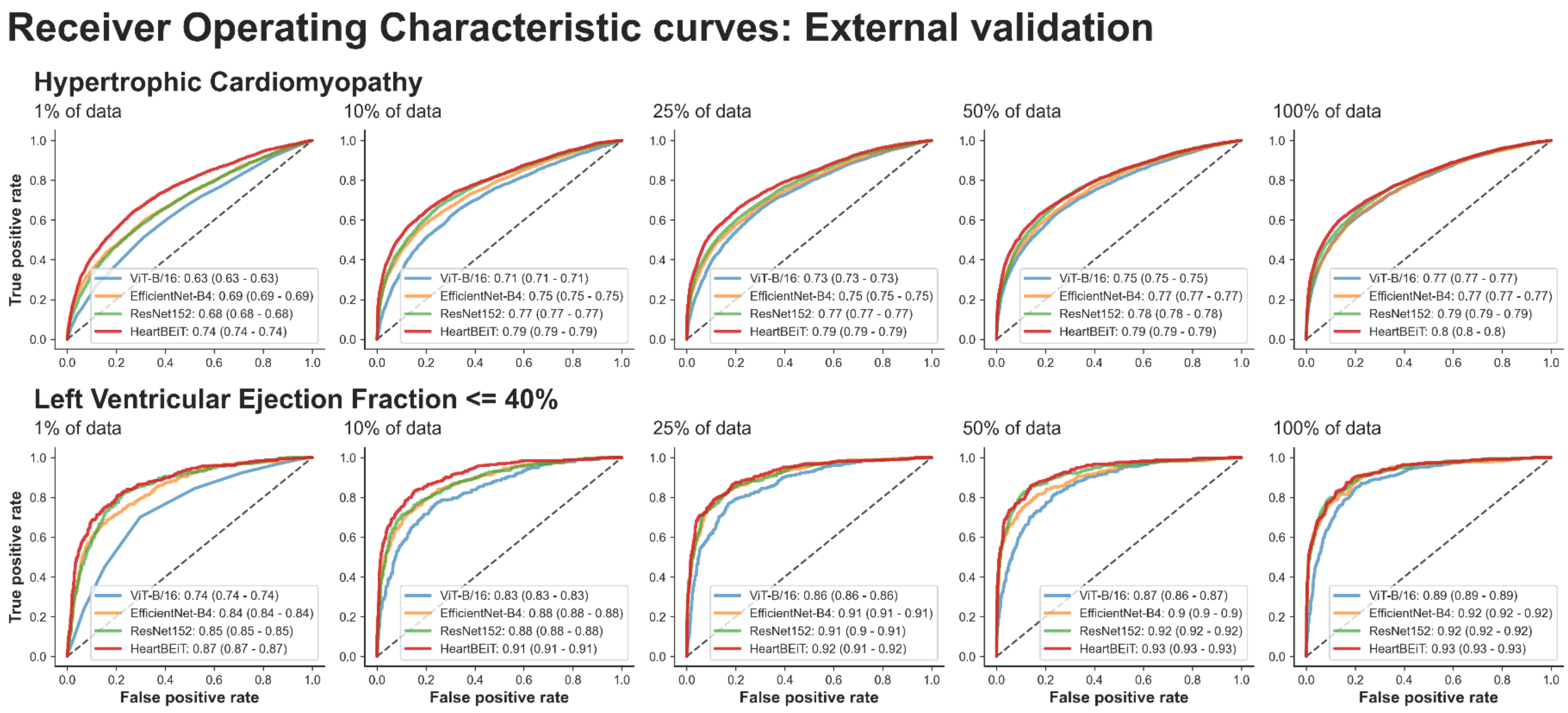

Supplementary Figure 3. Receiver Operating Characteristics Curves for External Validation of Vit-B/16, EfficientNet-B4, ResNet-152, and HeartBEiT for classification of Hypertrophic Cardiomyopathy, and Left Ventricular Ejection Fraction ≤40%.

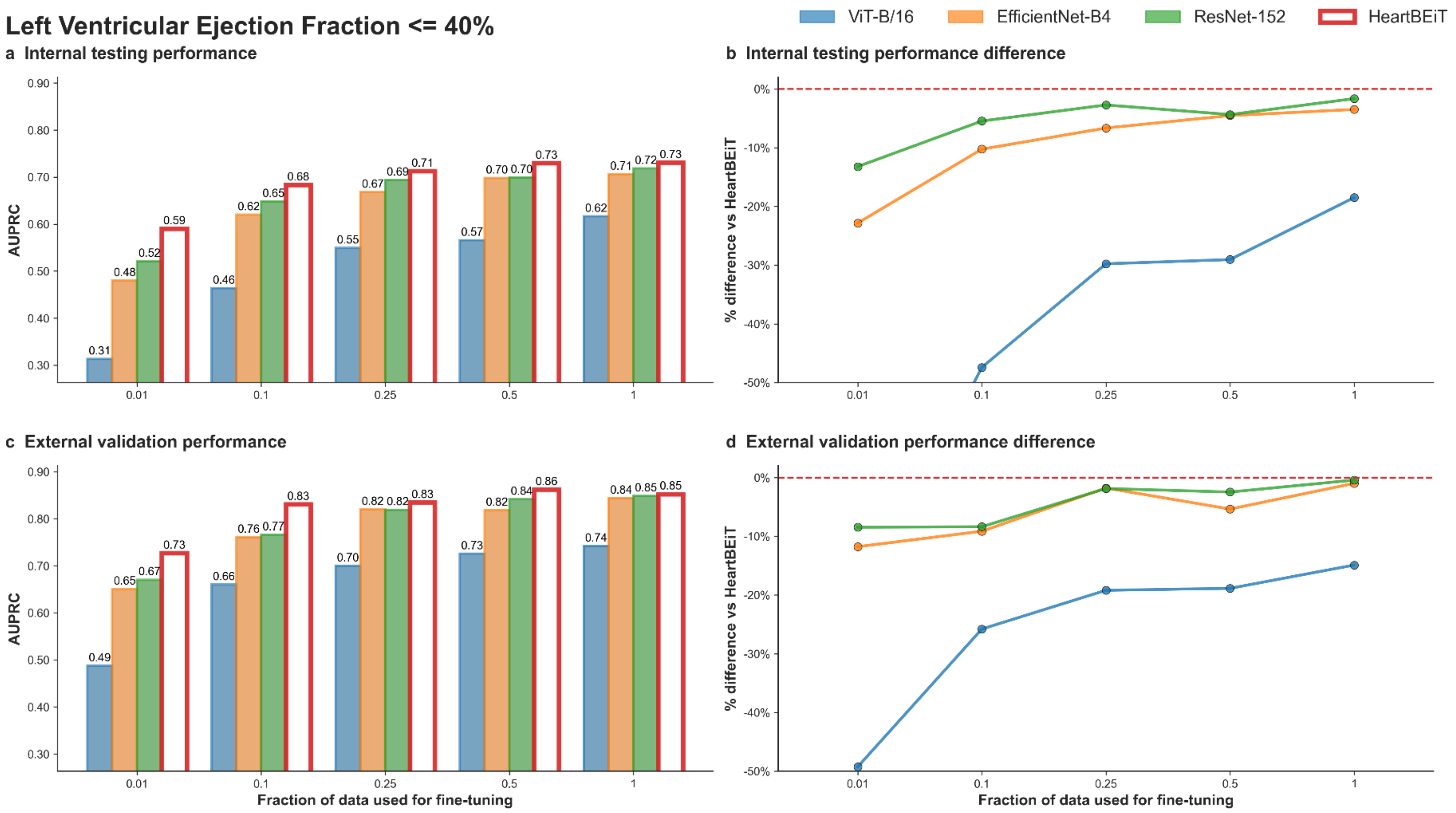

Supplementary Figure 4. Left Ventricular Ejection Fraction ≤ 40% classification on ECGs (Area Under Precision Recall Curve)

Panel a. Internal testing performance (4 Mount Sinai facilities)

Panel b. Internal testing performance difference

Panel c. External validation performance (Morningside patients)

Panel d. External validation performance difference

Red dashed lines in panels b and d indicate HeartBEiT performance.

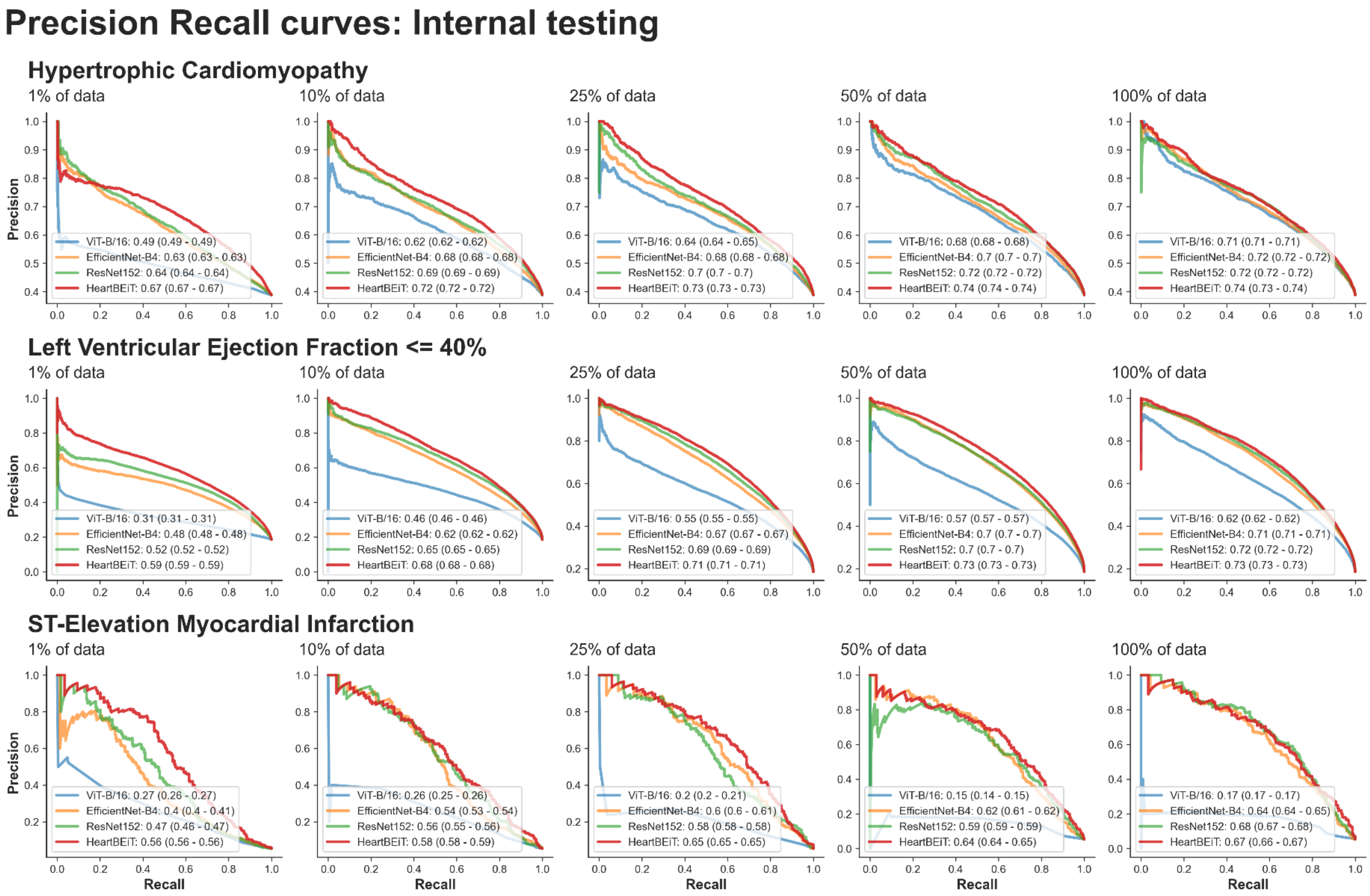

Supplementary Figure 5. Precision-Recall Curves for Internal Testing of Vit-B/16, EfficientNet-B4, ResNet-152, and HeartBEiT for classification of Hypertrophic Cardiomyopathy, Left Ventricular Ejection Fraction ≤40%, and ST-Elevation Myocardial Infarction.

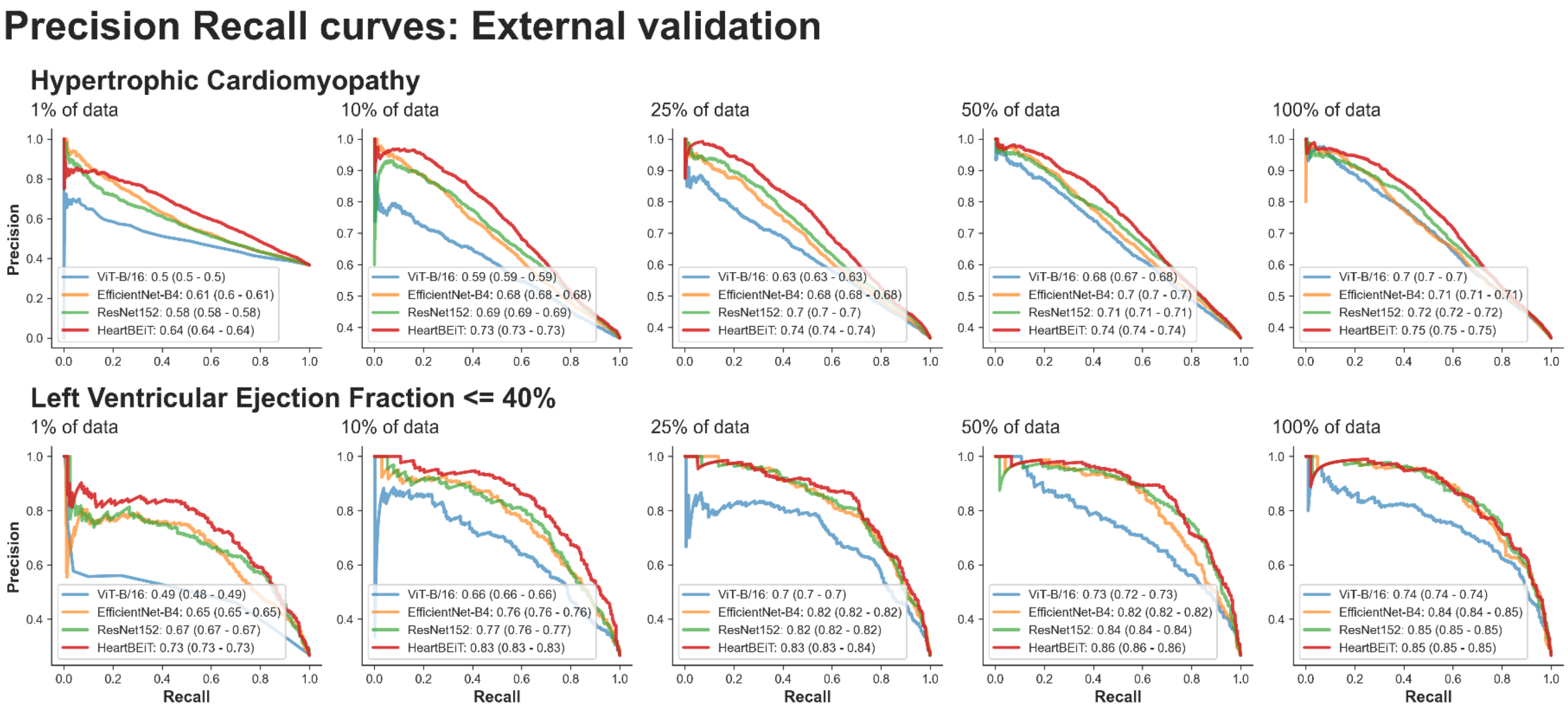

Supplementary Figure 6. Precision-Recall Curves for External Validation of Vit-B/16, EfficientNet-B4, ResNet-152, and HeartBEiT for classification of Hypertrophic Cardiomyopathy, and Left Ventricular Ejection Fraction ≤40%.

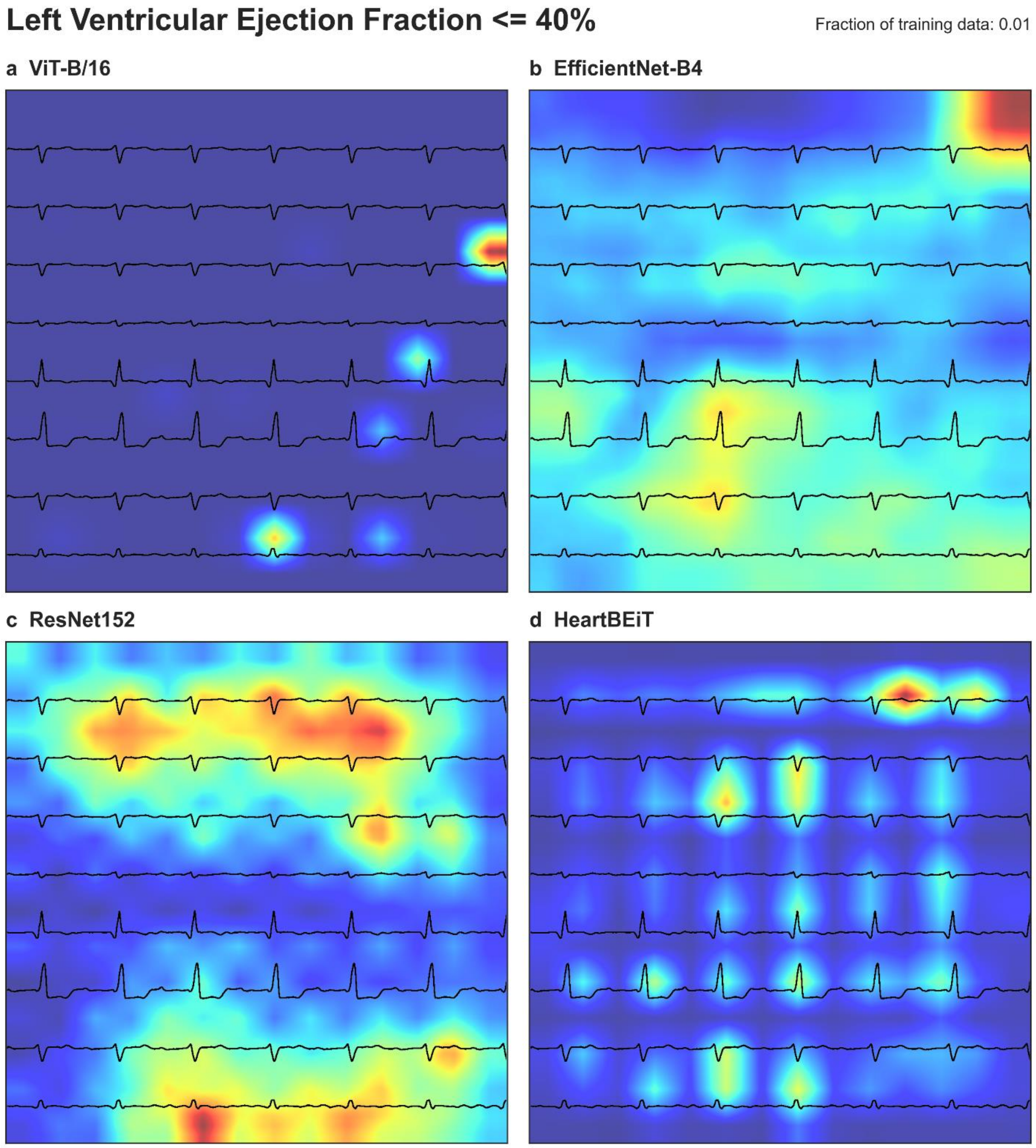

Supplementary Figure 7a. Gradient-weighted class activation saliency mapping images for ECG plots for classification of Left Ventricular Ejection Fraction ≤40% at 1% of training data.

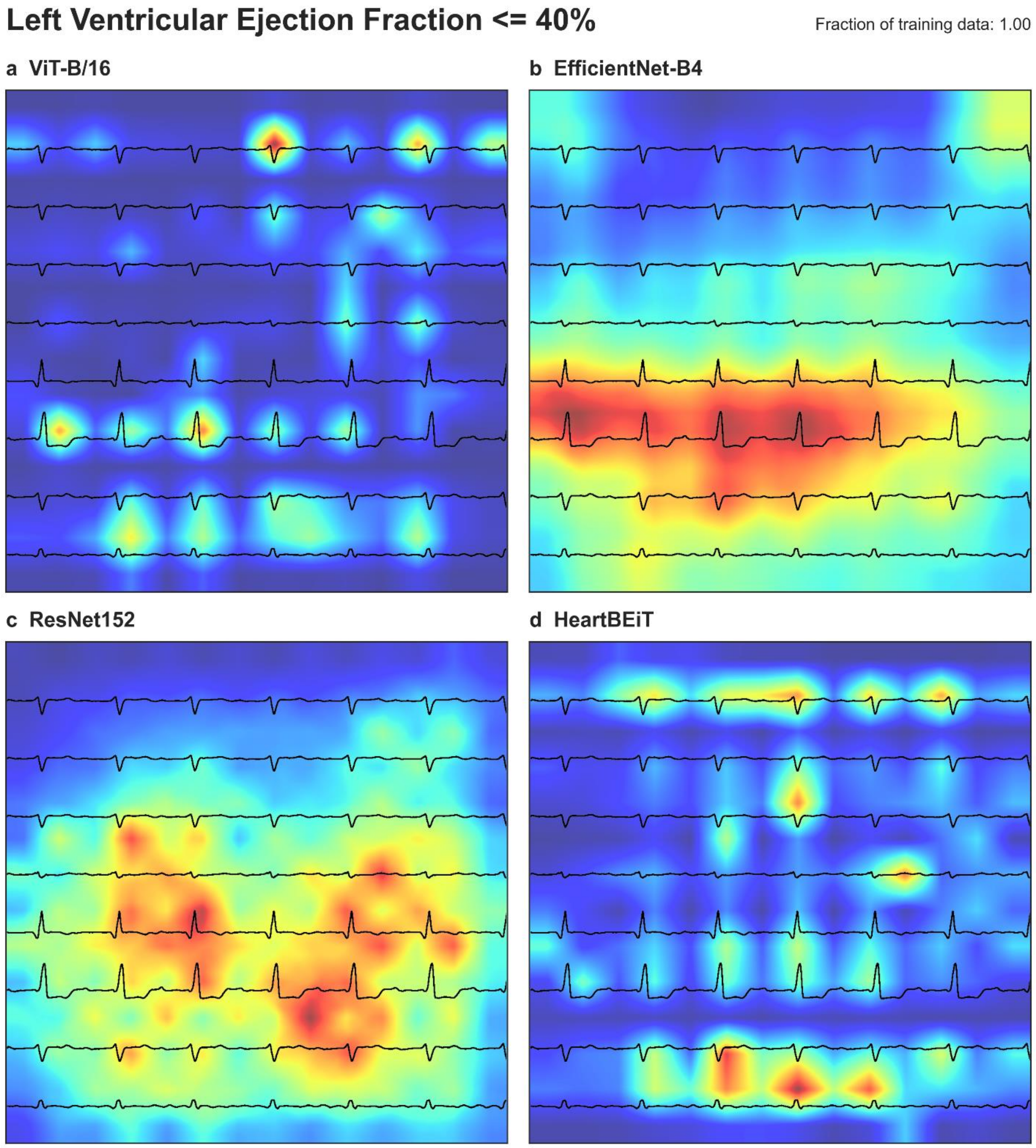

Supplementary Figure 7b. Gradient-weighted class activation saliency mapping images for ECG plots for classification of Left Ventricular Ejection Fraction ≤40% at 100% of training data.

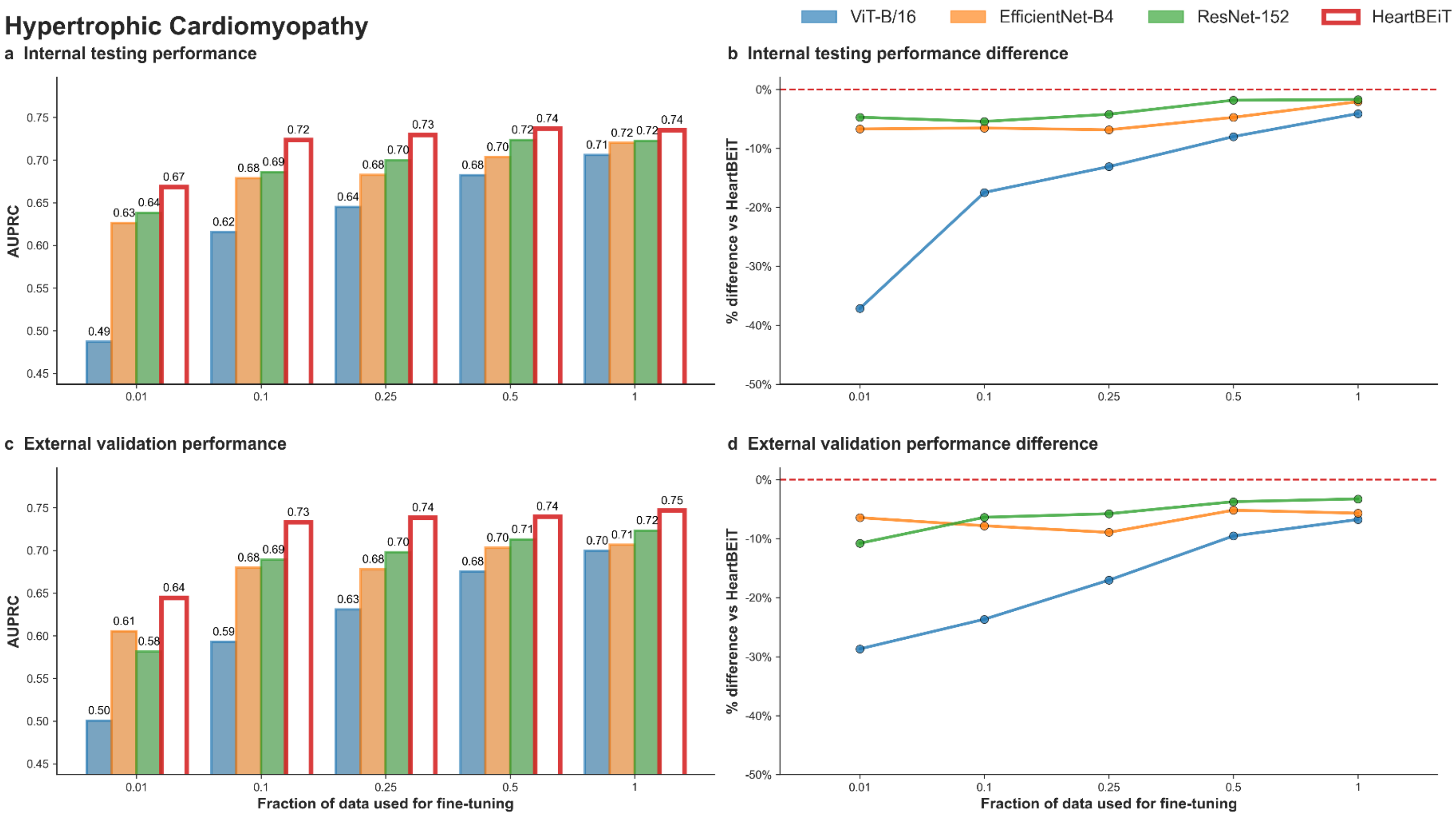

Supplementary Figure 8. Hypertrophic cardiomyopathy classification on ECGs (Area Under Precision Recall Curve)

Panel a. Internal testing performance (4 Mount Sinai facilities)

Panel b. Internal testing performance difference

Panel c. External validation performance (Morningside patients)

Panel d. External validation performance difference

Red dashed lines in panels b and d indicate HeartBEiT performance.

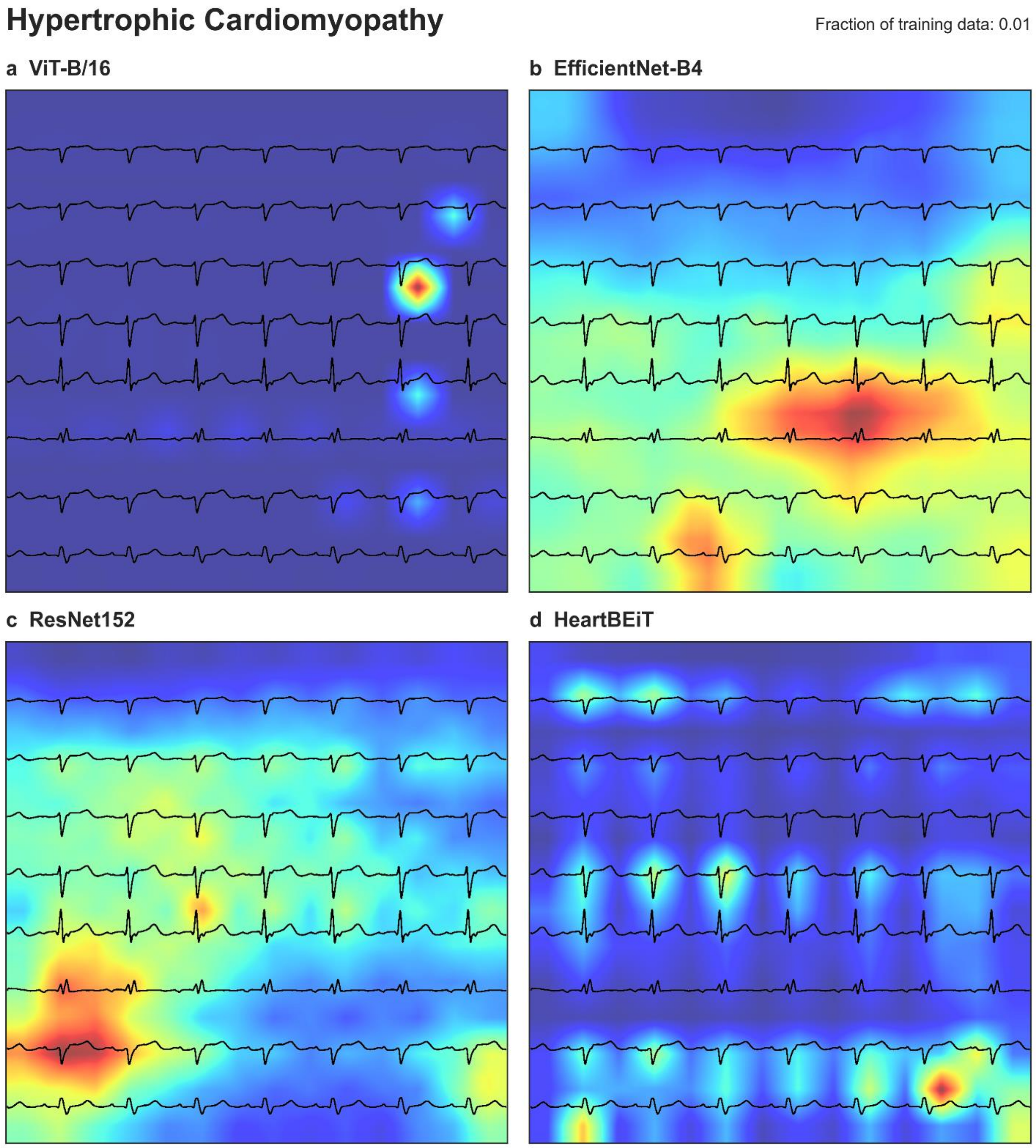

Supplementary Figure 9a. Gradient-weighted class activation saliency mapping images for ECG plots for classification of Hypertrophic Cardiomyopathy at 1% of training data.

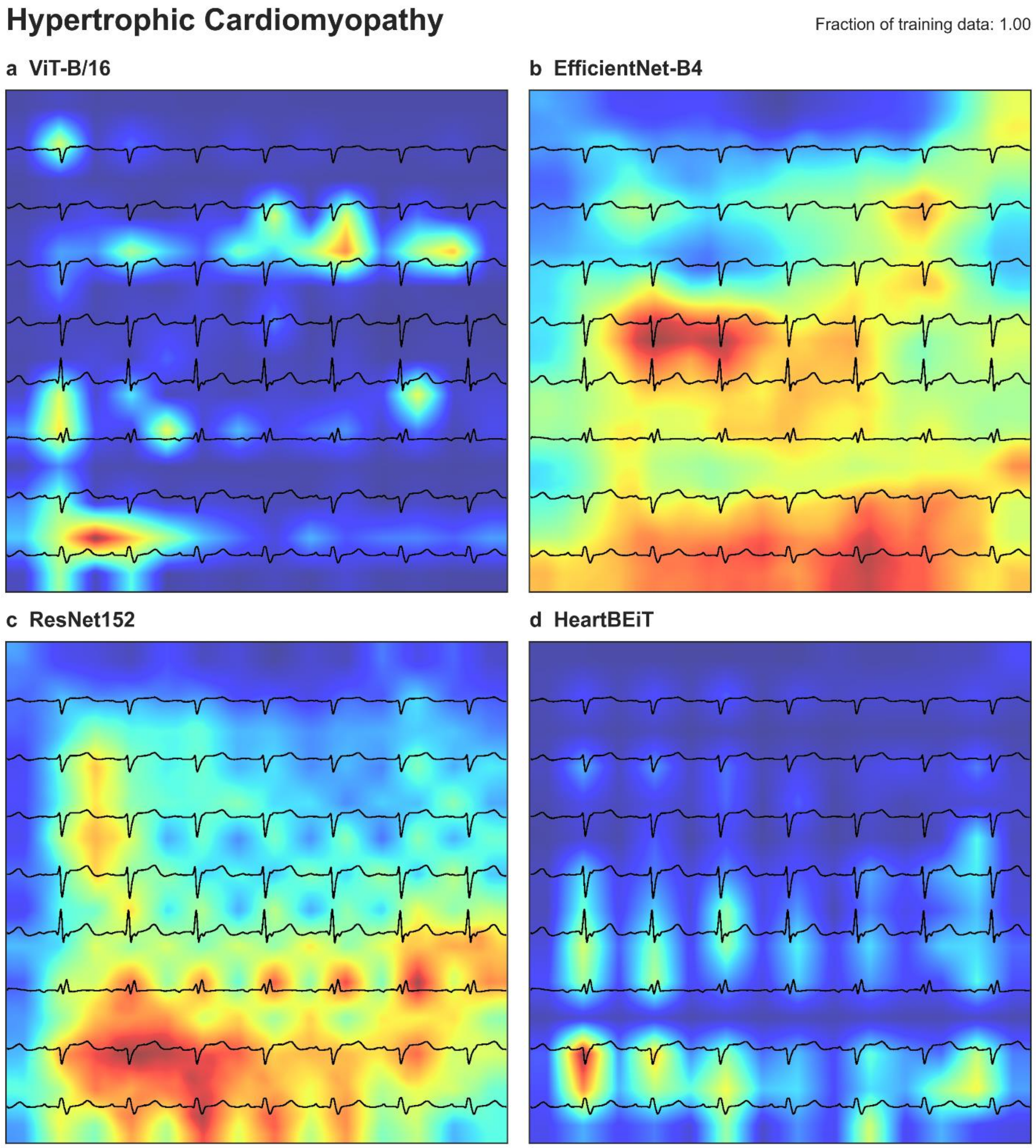

Supplementary Figure 9b. Gradient-weighted class activation saliency mapping images for ECG plots for classification of Hypertrophic Cardiomyopathy at 100% of training data.

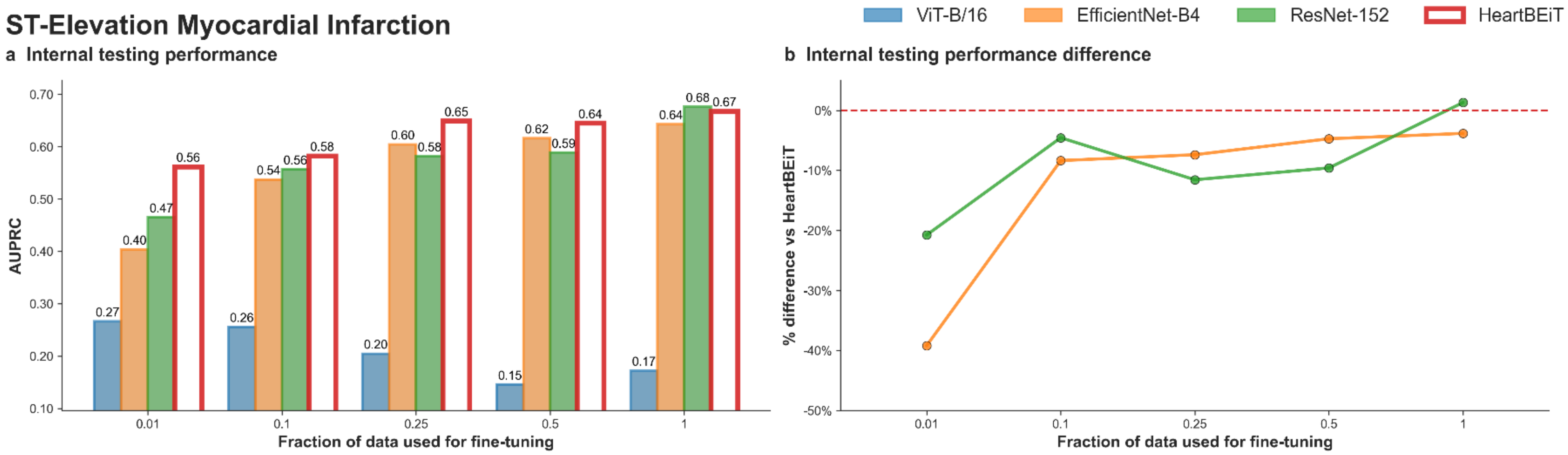

Supplementary Figure 10. STEMI detection on ECGs (PTB-XL database) (Areas Under Precision Recall Curve)
a. Internal testing performance
b. Internal testing performance difference
Red dashed line in panel b indicates HeartBEiT performance.

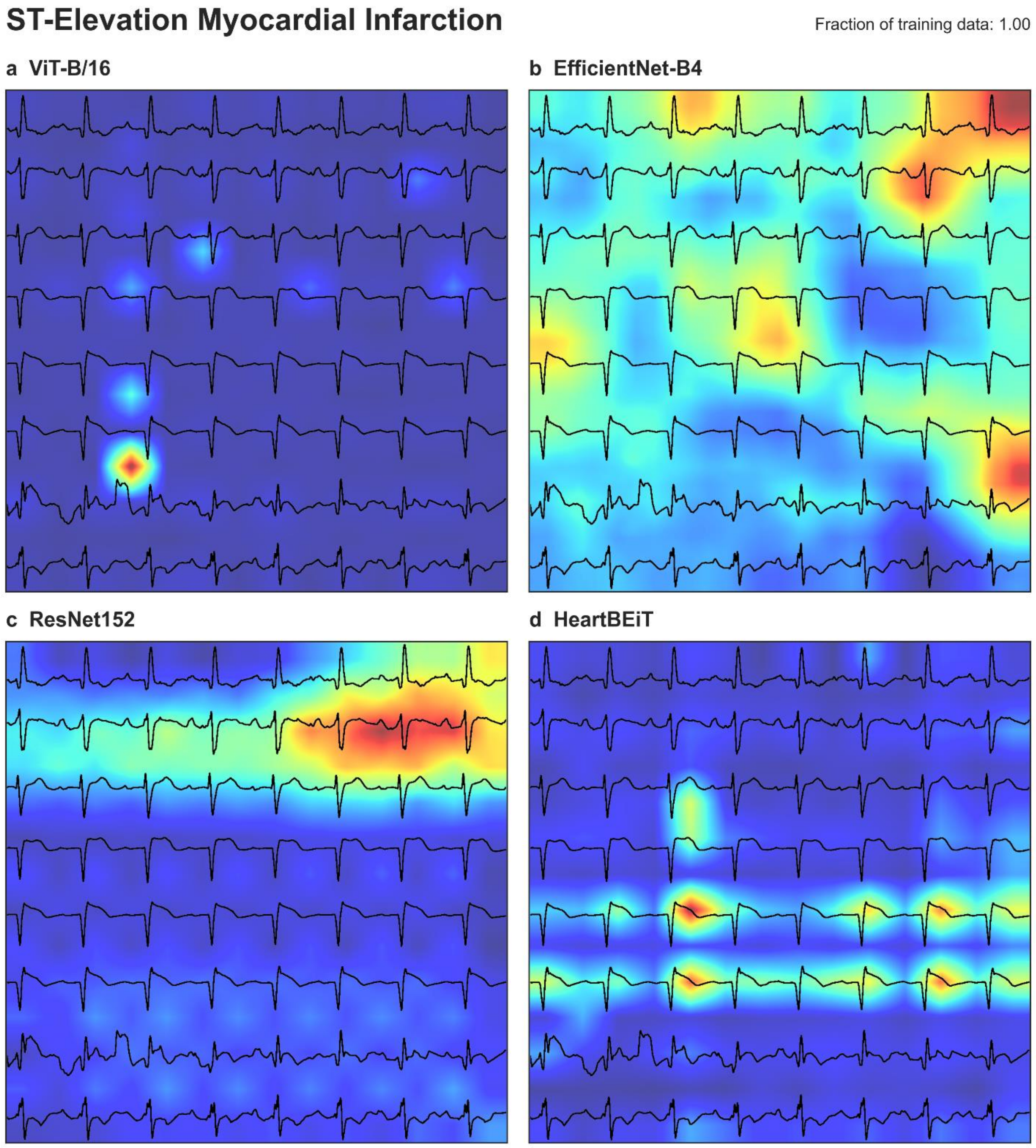

Supplementary Figure 11. Gradient-weighted class activation saliency mapping images for ECG plots for classification of ST-Elevation Myocardial Infarction at 100% of training data.

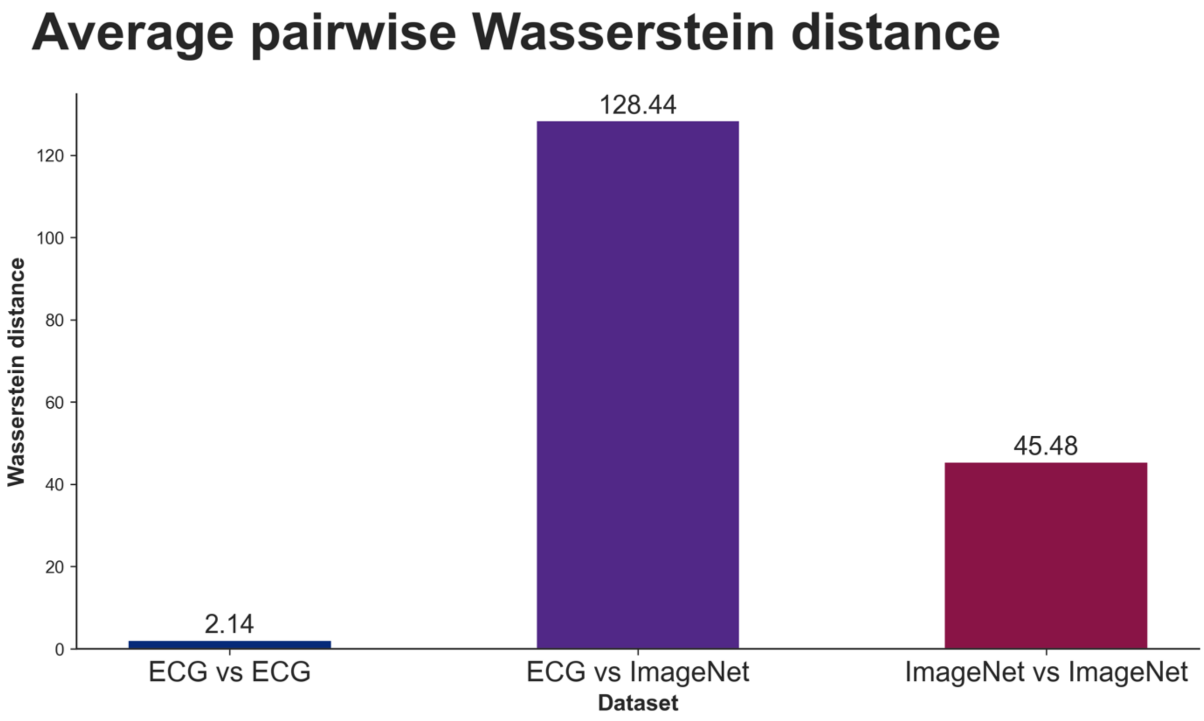

Supplementary Figure 12. Average pairwise Wasserstein distance across data modalities

Metric was calculated by randomly sampling 1000 ECGs and 1000 ImageNet images, and calculating the average pairwise Wasserstein distance between them, such that a total of 10^6 such calculations were made and averaged.

Supplementary Tables

	Model	0.01	0.1	0.25	0.5	1
	**Left ventricular ejection fraction <= 40%**					
Internal testing	ViT-B/16	0.67 (0.67 - 0.67)	0.80 (0.80 - 0.80)	0.84 (0.84 - 0.84)	0.85 (0.85 - 0.85)	0.86 (0.86 - 0.86)
	EfficientNet-B4	0.82 (0.82 - 0.82)	0.86 (0.86 - 0.86)	0.88 (0.88 - 0.88)	0.89 (0.89 - 0.89)	0.90 (0.90 - 0.90)
	ResNet-152	0.84 (0.84 - 0.84)	0.88 (0.88 - 0.88)	0.89 (0.89 - 0.89)	0.89 (0.89 - 0.89)	0.90 (0.90 - 0.90)
	HeartBEiT	***0.86 (0.86 - 0.86)***	***0.89 (0.89 - 0.89)***	***0.90 (0.90 - 0.90)***	***0.90 (0.90 - 0.90)***	***0.90 (0.90 - 0.90)***
	Fine-tuning samples	5,114	51,149	127,872	255,745	511,491
	Testing samples	128,687	128,687	128,687	128,687	128,687
External validation	ViT-B/16	0.74 (0.74 - 0.74)	0.83 (0.83 - 0.83)	0.86 (0.86 - 0.86)	0.87 (0.86 - 0.87)	0.89 (0.89 - 0.89)
	EfficientNet-B4	0.84 (0.84 - 0.84)	0.88 (0.88 - 0.88)	0.91 (0.91 - 0.91)	0.90 (0.90 - 0.90)	0.92 (0.92 - 0.92)
	ResNet-152	0.85 (0.85 - 0.85)	0.88 (0.88 - 0.88)	0.91 (0.90 - 0.91)	0.92 (0.92 - 0.92)	0.92 (0.92 - 0.92)
	HeartBEiT	***0.87 (0.87 - 0.87)***	***0.91 (0.91 - 0.91)***	***0.92 (0.91 - 0.92)***	***0.93 (0.93 - 0.93)***	***0.93 (0.93 - 0.93)***
	Testing samples	1,480	1,480	1,480	1,480	1,480
	**Hypertrophic Cardiomyopathy**					
Internal testing	ViT-B/16	0.61 (0.61 - 0.61)	0.72 (0.72 - 0.72)	0.75 (0.75 - 0.75)	0.77 (0.77 - 0.77)	0.79 (0.79 - 0.79)
	EfficientNet-B4	0.72 (0.72 - 0.72)	0.77 (0.77 - 0.77)	0.77 (0.77 - 0.77)	0.78 (0.78 - 0.79)	0.79 (0.79 - 0.79)
	ResNet-152	0.72 (0.72 - 0.72)	0.78 (0.78 - 0.78)	0.78 (0.78 - 0.78)	0.80 (0.80 - 0.80)	0.80 (0.80 - 0.80)
	HeartBEiT	***0.77 (0.77 - 0.77)***	***0.80 (0.80 - 0.80)***	***0.80 (0.80 - 0.80)***	***0.81 (0.81 - 0.81)***	***0.81 (0.81 - 0.81)***
	Fine-tuning samples	788	78,83	19,707	39,415	78,831
	Testing samples	20,448	20,448	20,448	20,448	20,448
External validation	ViT-B/16	0.63 (0.63 - 0.63)	0.71 (0.71 - 0.71)	0.73 (0.73 - 0.73)	0.75 (0.75 - 0.75)	0.77 (0.77 - 0.77)
	EfficientNet-B4	0.69 (0.69 - 0.69)	0.75 (0.75 - 0.75)	0.75 (0.75 - 0.75)	0.77 (0.77 - 0.77)	0.77 (0.77 - 0.77)
	ResNet-152	0.68 (0.68 - 0.68)	0.77 (0.77 - 0.77)	0.77 (0.77 - 0.77)	0.78 (0.78 - 0.78)	0.79 (0.79 - 0.79)
	HeartBEiT	***0.74 (0.74 - 0.74)***	***0.79 (0.79 - 0.79)***	***0.79 (0.79 - 0.79)***	***0.79 (0.79 - 0.79)***	***0.80 (0.80 - 0.80)***
	Testing samples	13,859	13,859	13,859	13,859	13,859
	**ST-Elevation Myocardial Infarction**					
Internal testing	ViT-B/16	0.80 (0.80 - 0.80)	0.81 (0.81 - 0.81)	0.79 (0.79 - 0.79)	0.77 (0.77 - 0.78)	0.79 (0.79 - 0.80)
	EfficientNet-B4	0.84 (0.83 - 0.84)	0.88 (0.88 - 0.88)	0.91 (0.91 - 0.91)	0.92 (0.91 - 0.92)	0.93 (0.93 - 0.93)

	ResNet-152	0.84<br>(0.84 - 0.84)	0.89<br>(0.89 - 0.89)	0.92<br>(0.92 - 0.92)	0.93<br>(0.93 - 0.93)	0.94<br>(0.94 - 0.94)
	HeartBEiT	***0.88<br>(0.88 - 0.89)***	***0.92<br>(0.92 - 0.92)***	***0.93<br>(0.93 - 0.93)***	***0.94<br>(0.94 - 0.94)***	***0.95<br>(0.95 - 0.95)***
	Fine-tuning samples	174	1,744	4,362	8,724	17,449
	Testing samples	4,352	4,352	4,352	4,352	4,352

Supplementary Table 1. Area Under the Receiver Operating Characteristic Curve (AUROC) metrics for various models compared to HeartBEiT at different fractions of fine-tuning and testing data.
Bold text indicates an outcome. Bold-italic text indicates highest performance at a task according to that performance metric.

	Model	0.01	0.1	0.25	0.5	1
	**Left ventricular ejection fraction <= 40%**					
Internal testing	ViT-B/16	0.31 (0.31 - 0.31)	0.46 (0.46 - 0.46)	0.55 (0.55 - 0.55)	0.57 (0.57 - 0.57)	0.62 (0.62 - 0.62)
	EfficientNet-B4	0.48 (0.48 - 0.48)	0.62 (0.62 - 0.62)	0.67 (0.67 - 0.67)	0.70 (0.70 - 0.70)	0.71 (0.71 - 0.71)
	ResNet-152	0.52 (0.52 - 0.52)	0.65 (0.65 - 0.65)	0.69 (0.69 - 0.69)	0.70 (0.70 - 0.70)	0.72 (0.72 - 0.72)
	HeartBEiT	***0.59 (0.59 - 0.59)***	***0.68 (0.68 - 0.68)***	***0.71 (0.71 - 0.71)***	***0.73 (0.73 - 0.73)***	***0.73 (0.73 - 0.73)***
	Fine-tuning samples	5,114	51,149	127,872	255,745	511,491
	Testing samples	128,687	128,687	128,687	128,687	128,687
External validation	ViT-B/16	0.49 (0.48 - 0.49)	0.66 (0.66 - 0.66)	0.70 (0.70 - 0.70)	0.73 (0.72 - 0.73)	0.74 (0.74 - 0.74)
	EfficientNet-B4	0.65 (0.65 - 0.65)	0.76 (0.76 - 0.76)	0.82 (0.82 - 0.82)	0.82 (0.82 - 0.82)	0.84 (0.84 - 0.85)
	ResNet-152	0.67 (0.67 - 0.67)	0.77 (0.76 - 0.77)	0.82 (0.82 - 0.82)	0.84 (0.84 - 0.84)	0.85 (0.85 - 0.85)
	**HeartBEiT**	**0.73 (0.73 - 0.73)**	**0.83 (0.83 - 0.83)**	**0.83 (0.83 - 0.84)**	**0.86 (0.86 - 0.86)**	**0.85 (0.85 - 0.85)**
	Testing samples	1,480	1,480	1,480	1,480	1,480
	**Hypertrophic Cardiomyopathy**					
Internal testing	ViT-B/16	0.49 (0.49 - 0.49)	0.62 (0.62 - 0.62)	0.64 (0.64 - 0.65)	0.68 (0.68 - 0.68)	0.71 (0.71 - 0.71)
	EfficientNet-B4	0.63 (0.63 - 0.63)	0.68 (0.68 - 0.68)	0.68 (0.68 - 0.68)	0.70 (0.70 - 0.70)	0.72 (0.72 - 0.72)
	ResNet-152	0.64 (0.64 - 0.64)	0.69 (0.69 - 0.69)	0.70 (0.70 - 0.70)	0.72 (0.72 - 0.72)	0.72 (0.72 - 0.72)
	HeartBEiT	***0.67 (0.67 - 0.67)***	***0.72 (0.72 - 0.72)***	***0.73 (0.73 - 0.73)***	***0.74 (0.74 - 0.74)***	***0.74 (0.73 - 0.74)***
	Fine-tuning samples	788	78,83	19,707	39,415	78,831
	Testing samples	20,448	20,448	20,448	20,448	20,448
External validation	ViT-B/16	0.50 (0.50 - 0.50)	0.59 (0.59 - 0.59)	0.63 (0.63 - 0.63)	0.68 (0.67 - 0.68)	0.70 (0.70 - 0.70)
	EfficientNet-B4	0.61 (0.60 - 0.61)	0.68 (0.68 - 0.68)	0.68 (0.68 - 0.68)	0.70 (0.70 - 0.70)	0.71 (0.71 - 0.71)
	ResNet-152	0.58 (0.58 - 0.58)	0.69 (0.69 - 0.69)	0.70 (0.70 - 0.70)	0.71 (0.71 - 0.71)	0.72 (0.72 - 0.72)
	HeartBEiT	***0.64 (0.64 - 0.64)***	***0.73 (0.73 - 0.73)***	***0.74 (0.74 - 0.74)***	***0.74 (0.74 - 0.74)***	***0.75 (0.75 - 0.75)***
	Testing samples	13,859	13,859	13,859	13,859	13,859
	**ST-Elevation Myocardial Infarction**					
Internal testing	ViT-B/16	0.27 (0.26 - 0.27)	0.26 (0.25 - 0.26)	0.20 (0.20 - 0.21)	0.15 (0.14 - 0.15)	0.17 (0.17 - 0.17)
	EfficientNet-B4	0.40 (0.40 - 0.41)	0.54 (0.53 - 0.54)	0.60 (0.60 - 0.61)	0.62 (0.61 - 0.62)	0.64 (0.64 - 0.65)
	ResNet-152	0.47 (0.46 - 0.47)	0.56 (0.55 - 0.56)	0.58 (0.58 - 0.58)	0.59 (0.59 - 0.59)	***0.68 (0.67 - 0.68)***

	HeartBEiT	***0.56 (0.56 - 0.56)***	***0.58 (0.58 - 0.59)***	***0.65 (0.65 - 0.65)***	***0.64 (0.64 - 0.65)***	0.67 (0.66 - 0.67)
	Fine-tuning samples	174	1,744	4,362	8,724	17,449
	Testing samples	4,352	4,352	4,352	4,352	4,352

Supplementary Table 2. Area Under Precision Recall Curve (AUPRC) metrics for various models compared to HeartBEiT at different fractions of fine-tuning and testing data.
Bold text indicates an outcome. Bold-italic text indicates highest performance at a task according to that performance metric.
